# Supplementary material for: Atmospheric hydrogen consumption is regulated by glycerol-mediated catabolite repression in mycobacteria
Source: mSystems. 2026 Mar 16;11(4):e01678-25. doi: 10.1128/msystems.01678-25 (PMC13098282; doi:10.1128/msystems.01678-25)
Supplement: Supplemental Material — Supplemental tables and figures. [file msystems.01678-25-s0001.docx]

**Supplementary material**

**Table S1. Bacterial strains and plasmids used in this study.**

| **Strain** | **Description** | **Reference** |
| --- | --- | --- |
| mc^2^155 | Wildtype strain of *Mycobacterium smegmatis* | (1) |
| *gylR* mutant | Isogenic strain to WT, but possesses a frameshift mutation (Leu154) to *MSMEG_6757* (*gylR*) | This study |
| WT + pMV261 | WT strain with empty pMV261 for complementation assay | This study |
| *gylR* mutant + pMV261 | *gylR* mutant strain with empty pMV261 for complementation assay | This study |
| *gylR* mutant + p(*gylR*) | *gylR* mutant strain with pMV261(*gylR*) for complementation assay | This study |
| *gylR* knockdown | WT strain with pLJR962_KDgylR | This study |
| DH5α | *Escherichia coli* F– φ80*lacZ*ΔM15 Δ(*lacZ*YA*argF*)U169 *recA*1 *endA*1 *hsdR*17(rK–, mK+) *phoA* *supE*44 λ– *thi*-1 *gyrA*96 *relA*1 | Thermo Fischer |
| C41 (DE3) | Standard *Escherichia coli* lab strain used for recombinant protein expression | Thermo Fischer |
| **Plasmids** | **Description** | **Reference** |
| pMV261 | Kan^r^, mycobacterial oriM, pBR322 ori, P_hsp60_ promoter | (2) |
| p(*gylR*) | pMV261 containing *MSMEG_6757* (*gylR*) gene insert for *gylR* mutant complementation | This study |
| pET-23a | Protein expression vector possessing N-terminal T7-Tag and Amp^r^ | Novagen |
| pET-23a(*gylR*) | pET-23a containing *gylR* sequence for recombinant protein expression. | This study |
| pET-23a(*crp1*) | pET-23a containing *crp1* sequence for recombinant protein expression. | This study |
| pET-23a(*crp2*) | pET-23a containing *crp2* sequence for recombinant protein expression. | This study |
| pLJR962 | Sth1 dCas9; Sth1 sgRNA scaffold; Tet repressor; L5- integrating backbone; ColE1 ori (E. coli); Kan^r^ | (3) |
| pLJR962_KDgylR | pLJR962 with sgRNA targeting *gylR* for repression | This study |

**Table S2. Primers used in this study.**

| **Primers** | **Sequence (5’ to 3’)** | **Purpose** |
| --- | --- | --- |
| hucp_fw | CGACCAGACGCGCGGCCTC | Amplification of *huc* promoter of EMSA |
| hucp_rev | GACCGGCGAGATGTCTGGAAGTTC |  |
| glpp_fw | GTAGCTGCAGTATCGCCGCGG | Amplification of *glpFKD* promoter of EMSA |
| glpp_rev | CACCCCGAACAGGATGAGGATGC |  |
| gylrC_fw | GTCAGGGATCCATGCCAGGCACTGTGCAGTCCGTG | Amplification of *gylR* for complementation vector construction |
| gylrC_rev | GTCAGAAGCTTTCACAGTTCCCGCCCGTGGC |  |
| gylR_fw | GTCAGCCATGGGTCCAGGCACTGTGCAGTCCGTG | Amplification of *gylR* for recombinant protein expression |
| gylR_rev | GTCAGCTCGAGCAGTTCCCGCCCGTGGCC |  |
| crp1_fw | GTCAGCCATGGGTGACGAAGTGCTGGCGCGCG | Amplification of *crp1* for recombinant protein expression |
| crp1_rev | GTCAGAAGCTTTCAGTTCGCGCGCCGCGC |  |
| crp2_fw | GTCAGCCATGGGTGACGAGATCCTGGCCAGGGC | Amplification of *crp2* for recombinant protein expression |
| crp2_rev | GTCAGAAGCTTCTAGCGGGCGCGGCGGGC |  |
| gylr_KD_fw | GGGAATCCAGTTGGGTCGAACCGAG | sgRNA targeting *gylR* repression |
| gylr_KD_rev | AAACCTCGGTTCGACCCAACTGGAT |  |
| pljr962_fwd | GCTCTTCAGGATCTGACCAGGGAAAATAGCCCTC | Screening of strains with pLJR962_KDgylR |
| pljr962_rev | GCTCTTCACTGAAAAAAATAAAAAAGGGGACCTCTA |  |

**Table S3 (xlsx). Summary of proteomic analysis data.** Spreadsheet details the results of the shotgun proteomic experiment, which compared the relative abundance of proteins in the gylR mutant strain vs WT M. smegmatis, as well as a gylR knockdown vs WT M. smegmatis, at exponential phase and stationary phase. The spreadsheet also contains known M. smegmatis proteins mapped to KEGG pathways and modules, with annotations assigned to proteins identified in the gylR mutant and WT strains.

**
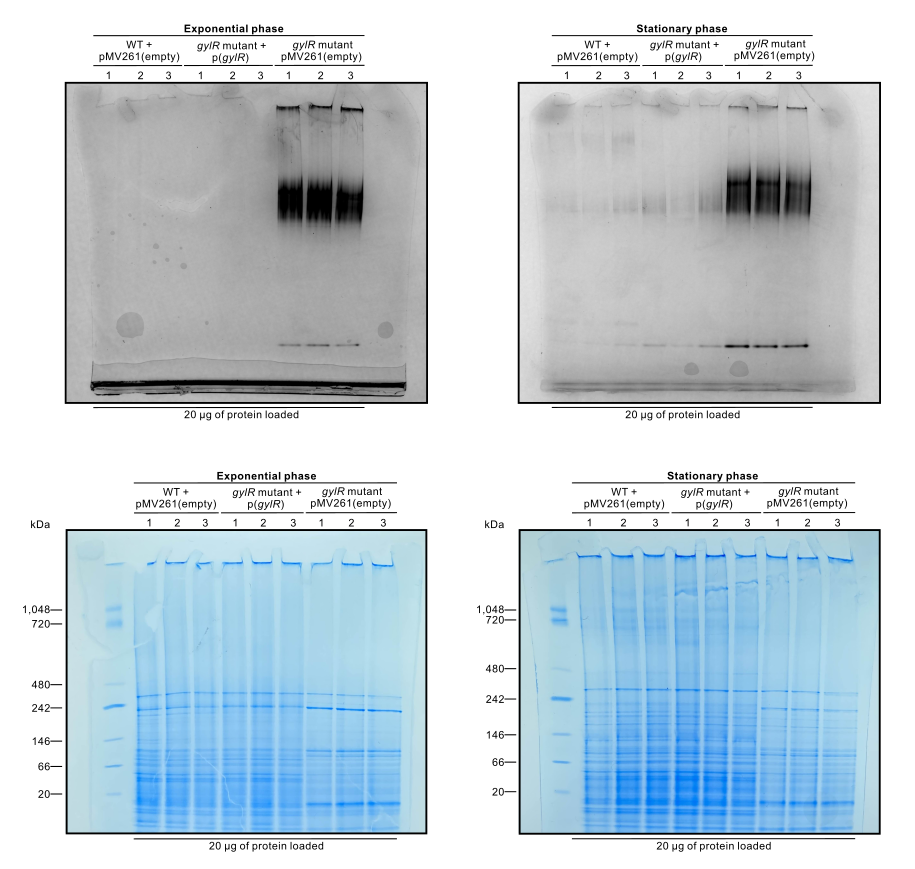
**

**Figure S1. Native-PAGE hydrogenase activity staining of the *gylR* mutant** **frameshift mutant**. Native gels depicting Huc hydrogenase activity stained with the artificial electron acceptor NBT (top) and Coomassie gel stained using AcquaStain (bottom). WT *M. smegmatis* (WT + pMV261(empty), *gylR* mutant (*gylR* mutant + pMV261(empty) and the *gylR* mutant complementation strain (*gylR* mutant*:*p(*gylR*)) were harvested in triplicate (n=3) at exponential phase (OD_600_ = 1.4-1.6) and stationary phase (OD_max_ + 1 day) for activity quantification using densitometry. 20 µg of each sample was loaded.


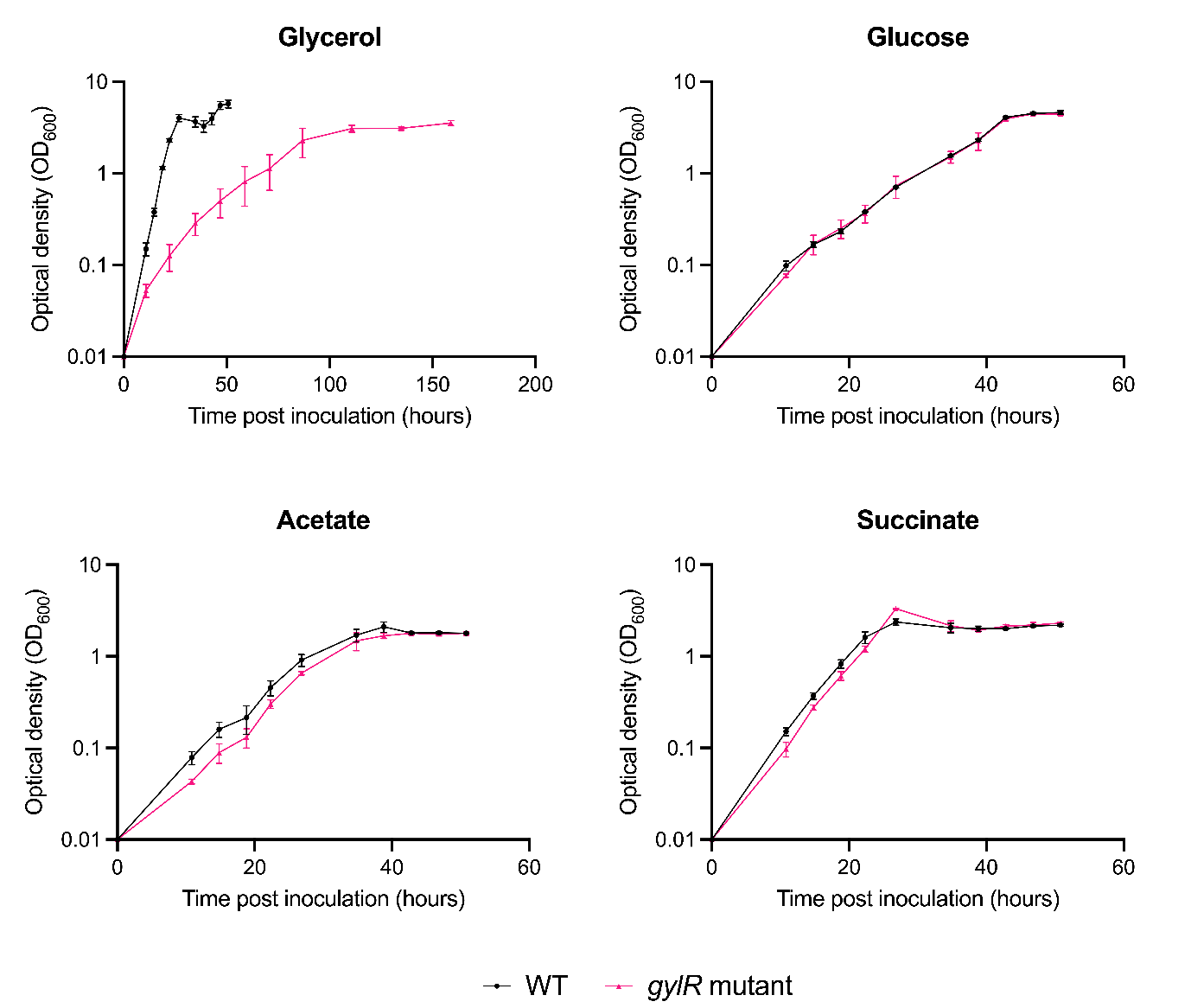


**Figure S2. The presence of a non-functional GylR does not impact the growth of *M. smegmatis* with alternative organic substrates.** Comparative growth of WT *M. smegmatis* and the *gylR* frameshift mutant grown in minimal media supplemented with 0.2% of one of four carbon sources: glycerol, glucose, acetate or succinate. Pre-cultures were grown in LB media overnight, then a small volume of culture was diluted into the new minimal media (with additional carbon source) upon inoculation. Error bars demonstrate the standard deviations of three (n=3) biological replicates.


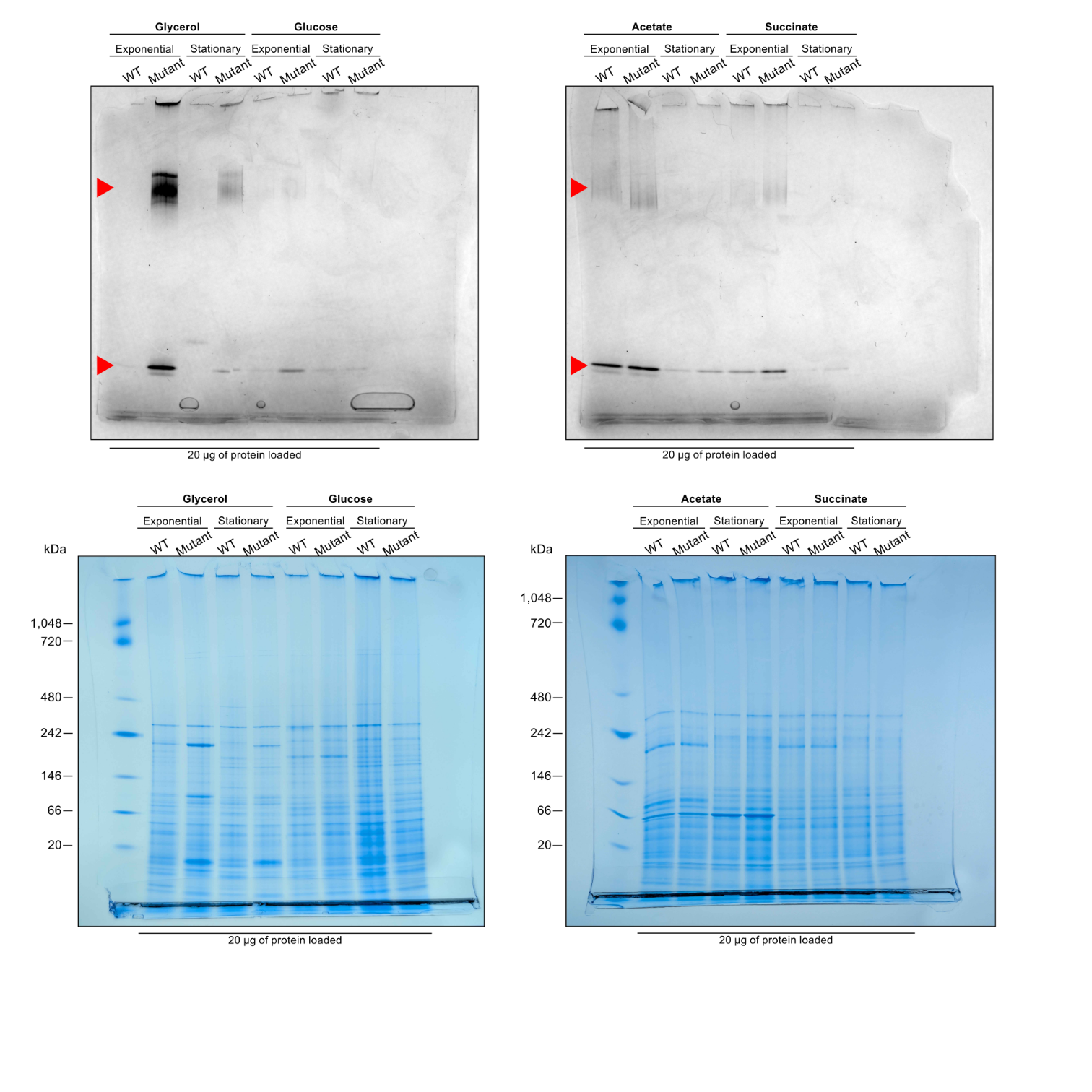


**Figure S3. Huc activity in WT *M. smegmatis* and *gylR* mutant strains** **when grown with different carbon sources.** Native gels depicting hydrogenase activity stained with NBT (top) and Coomassie gel stained with AcquaStain (bottom). Strains were grown with either 0.2% glycerol, glucose, acetate or succinate, with cultures harvested at exponential phase (OD_600_ = 1.3-1.5 for growth with glycerol, OD_600_ = 1.45 for growth with glucose, OD_600_ = 1.2 for growth with succinate, and OD_600_ = 0.9 for growth with acetate) and stationary phase (OD_max_ + 1 day). Red arrows indicate oligomeric Huc staining (upper) and dimeric Huc staining (lower).


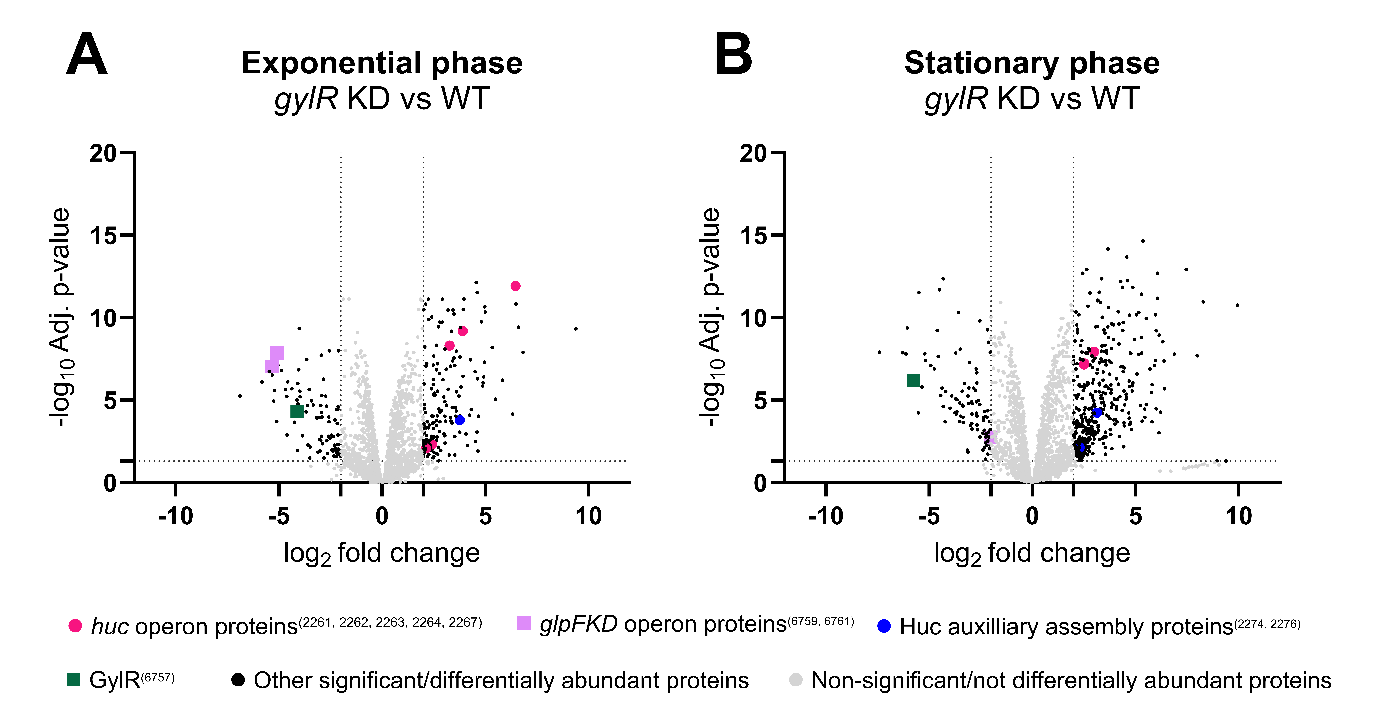


**Figure S4: Comparative proteomic analysis of a CRISPRi-generated knockdown of *gylR* and WT *M. smegmatis*.** Comparative shotgun proteomics volcano plots demonstrating the differential abundance of proteins in the *gylR* knockdown compared to WT *M. smegmatis* when grown with glycerol as the sole carbon source at exponential phase (OD_600_ = 1.5) **(A)** and carbon-depleted stationary phase (OD_max_ + 1 day) **(B)**.


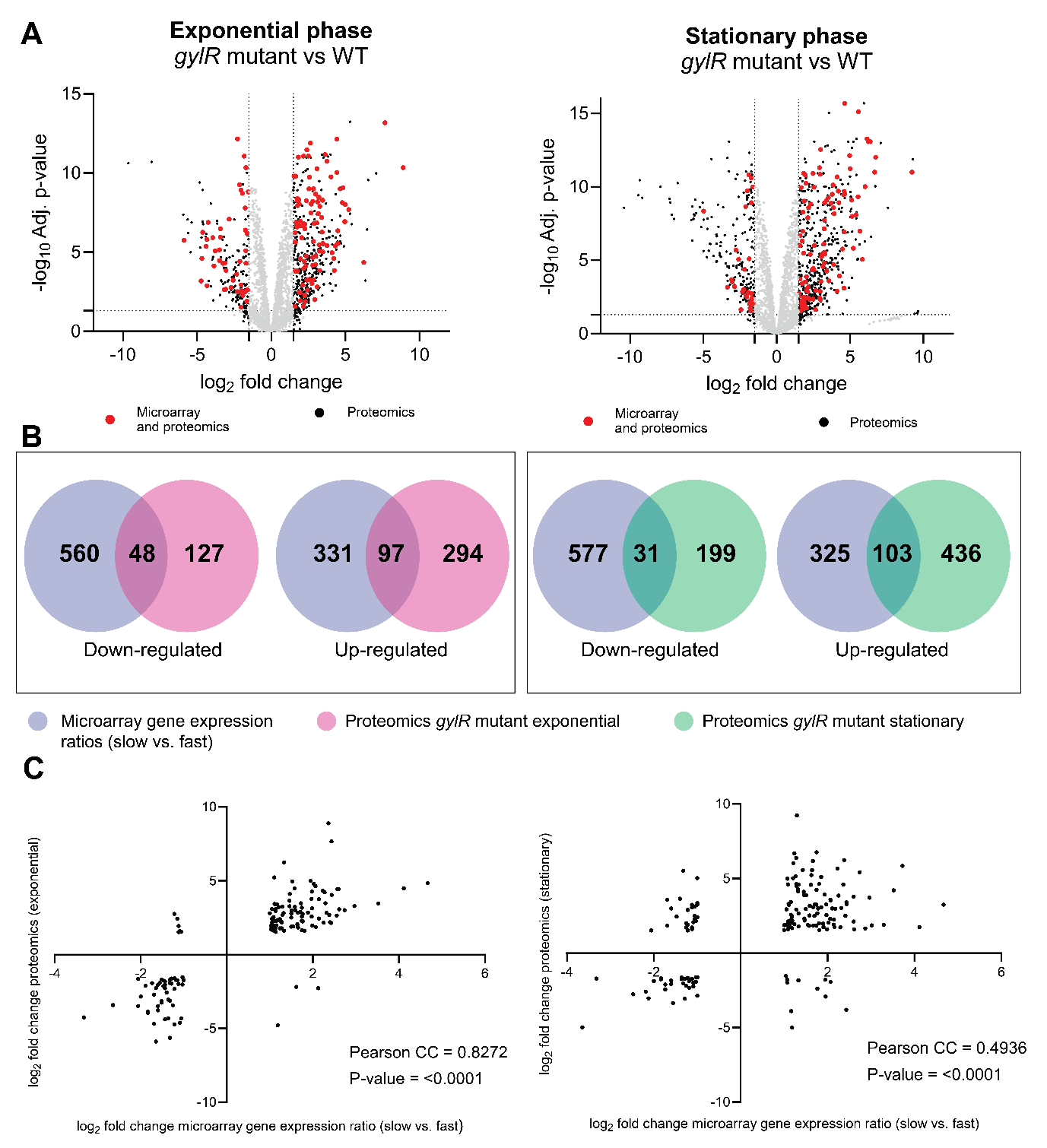


**Figure S5: A comparison of genes differentially expressed in *M. smegmatis* cells during slow vs. fast growth in continuous culture on glycerol and the *gylR* mutant proteomics (A)** Comparative shotgun proteomics volcano plots demonstrating the differential abundance of proteins in the *gylR* mutant compared to WT *M. smegmatis* when grown with glycerol as the sole carbon source at exponential phase (OD_600_ = 1.5) and carbon-depleted stationary phase (OD_max_ + 1 day). Proteins with statistically significant differences in abundance (p < 0.05, log_2_ FC ≥ 1.5, log_2_ FC ≤ -1.5) are represented by the dark-coloured dots, and those that are not significant are in grey. Red dots indicate proteins that were also found to be up- or down-regulated in microarray data of differentially expressed genes during slow vs. fast growth in continuous culture on glycerol . **(B)** Venn diagrams showing the number of genes significantly up- and down-regulated during slow vs fast growth in continuous culture on glycerol (from microarray data) (blue circle) (4) and proteins with differential abundance in the *gylR* mutant at exponential phase (pink circle) or stationary phase (proteomics data) (green circle). **(C)** Scatter plots comparing changes in protein abundance in the gylR mutant with gene expression-level changes reported in the continuous-culture dataset (4). Only genes/proteins differentially expressed in both datasets are shown. A stronger correlation is observed during exponential growth than in the stationary phase, indicating that the proteomic effects of gylR loss more closely mirror transcriptional responses to low glycerol availability during active growth.


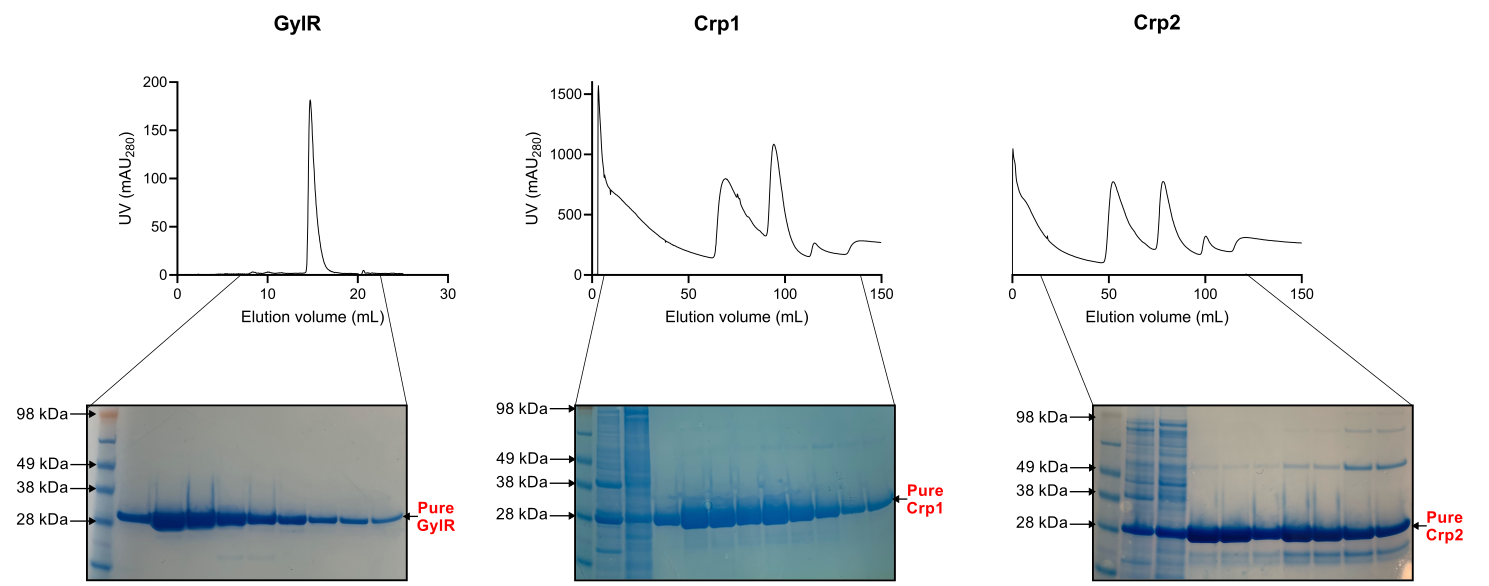


**Figure S6. Purification of recombinantly-expressed GylR, Crp1 and Crp2.** Upper panel: Chromatograms depicting elution profile of GylR (SEC), Crp1 (affinity chromatography) and Crp2 (affinity chromatography). Lower panel: SDS-PAGE gels containing the fractions corresponding to the chromatogram peaks.


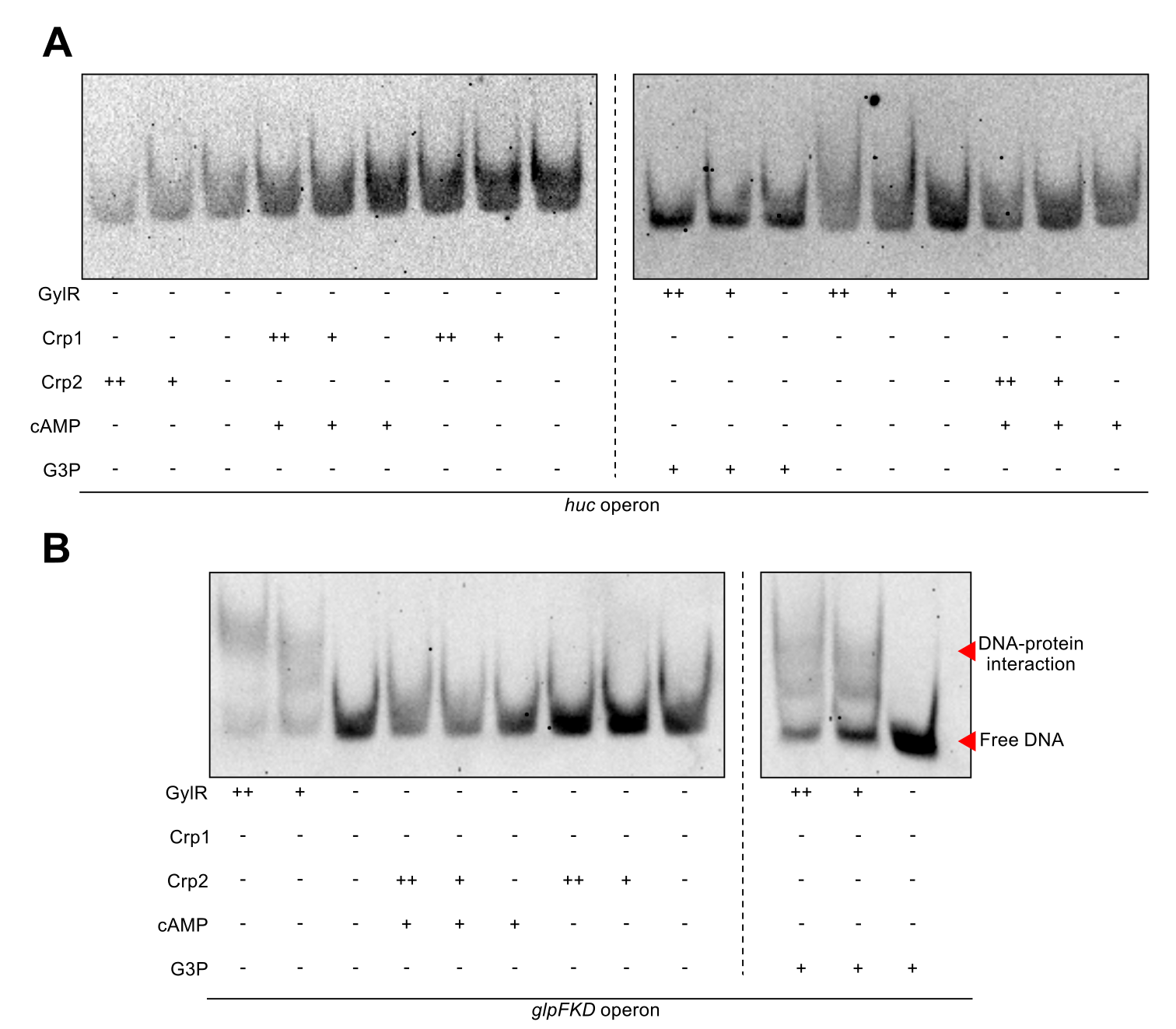


**Figure S7. Electrophoretic Mobility Shift Assays investigating the binding of GylR and CRP to the huc promoter region.** Electrophoretic mobility shift assays depicting the binding of GylR and CRP homologues Crp1 and Crp2 to the huc **(A)** and glpFKD **(B)** operon promoter regions (5, 6). GylR and Crp1/2 were added at a concentration of either 3 µM (++) or 1 µM (+), with cAMP and G3P added to potentially stimulate protein binding at respective concentrations of 50 mM. The binding of GylR to the glpFKD promoter was used as a positive control, with DNA-protein interaction indicated by the upward shift in band molecular weight as protein concentration increases.

**References**

1. Snapper SB, Melton RE, Mustafa S, Kieser T, Jr WRJ. 1990. Isolation and characterization of efficient plasmid transformation mutants of *Mycobacterium smegmatis*. Mol Microbiol 4:1911-1919.

2. Stover CK, de la Cruz VF, Fuerst TR, Burlein JE, Benson LA, Bennett LT, Bansal GP, Young JF, Lee MH, Hatfull GF, Snapper SB, Barletta RG, Jacobs WR, Bloom BR. 1991. New use of BCG for recombinant vaccines. Nature 351:456-460.

3. Rock JM, Hopkins FF, Chavez A, Diallo M, Chase MR, Gerrick ER, Pritchard JR, Church GM, Rubin EJ, Sassetti CM, Schnappinger D, Fortune SM. 2017. Programmable transcriptional repression in mycobacteria using an orthogonal CRISPR interference platform. Nat Microbiol 2:16274.

4. Berney M, Cook GM. 2010. Unique flexibility in energy metabolism allows mycobacteria to combat starvation and hypoxia. PLoS One 5:e8614.

5. Berney M, Greening C, Hards K, Collins D, Cook GM. 2014. Three different [NiFe] hydrogenases confer metabolic flexibility in the obligate aerobe *Mycobacterium smegmatis*. Environ Microbiol 16:318-30.

6. Bong HJ, Ko EM, Song SY, Ko IJ, Oh JI. 2019. Tripartite Regulation of the *glpFKD* Operon Involved in Glycerol Catabolism by GylR, Crp, and SigF in *Mycobacterium smegmatis*. J Bacteriol 201.
